# Feasibility and Initial Performance of Simultaneous SPECT-CT Imaging Using a Commercial Multi-Modality Preclinical Imaging System

**DOI:** 10.1155/2015/134768

**Published:** 2015-06-03

**Authors:** Dustin R. Osborne, Derek W. Austin

**Affiliations:** ^1^Graduate School of Medicine, University of Tennessee, Knoxville, TN 37920, USA; ^2^Welch Allyn, Knoxville, TN 37923, USA

## Abstract

Multi-modality imaging provides coregistered PET-CT and SPECT-CT images; however such multi-modality workflows usually consist of sequential scans from the individual imaging components for each modality. This typical workflow may result in long scan times limiting throughput of the imaging system. Conversely, acquiring multi-modality data simultaneously may improve correlation and registration of images, improve temporal alignment of the acquired data, increase imaging throughput, and benefit the scanned subject by minimizing time under anesthetic. In this work, we demonstrate the feasibility and procedure for modifying a commercially available preclinical SPECT-CT platform to enable simultaneous SPECT-CT acquisition. We also evaluate the performance of simultaneous SPECT-CT tomographic imaging with this modified system. Performance was accessed using a ^57^Co source and image quality was evaluated with ^99m^Tc phantoms in a series of simultaneous SPECT-CT scans.

## 1. Introduction

This work examines a simultaneous acquisition method for combined Single Photon Emission Computed Tomography (SPECT) and X-ray Computed Tomography (CT) imaging systems. Currently all commercially available SPECT-CT systems for preclinical imaging use a sequential scan mode that results in undesirably long acquisition times that limit the potential throughput of the imaging system. Acquisition of simultaneous multi-modality data may improve temporal and physiological correlation between each modality and improve image registration. Additionally, reducing scan times minimizes the time any given animal must remain under anesthesia.

Although SPECT scans typically demand the greatest time requirements, complementary CT scans in a preclinical system can add roughly 2 to 15 minutes or more depending upon the imaging system and the selected protocol. These scan times are significantly higher than those found on clinical gantries as most preclinical imaging systems lack slip ring technology that enables high speed rotation and true helical acquisition.

Here, we look at the possibility of modifying a* commercially available* Inveon preclinical SPECT-CT system (Siemens Medical Solutions USA, Inc., Knoxville, TN) such that both modalities acquire data simultaneously, thus reducing the overall acquisition time. Whereas previous work by Nagarkar et al. worked to develop a custom-designed system with the capability of performing simultaneous SPECT-CT, the system was only available as a resource to a single research group [1]. In contrast, this work sought to use a commercially available system with minor modifications to the system in order to develop new functionality that may be accessible to a larger number of institutions worldwide. In this work, we address key considerations that may allow similar principles as those described for our available imaging equipment to be applied to a number of other commercial platforms both preclinically and clinically.

Several benefits exist to performing simultaneous SPECT-CT imaging. The acquisition of sequential SPECT-CT data always suffers from temporal separation [2]. This results in a mismatch between anatomical and physiological data. Simultaneous acquisition of SPECT and CT modalities enables improved temporal matching and the potential for more accurate image registration that may impact the application of attenuation and scatter correction. Simultaneous imaging with respiratory or cardiac monitoring hardware would enable the use of the same physiological signal for each modality for use in correcting this motion.

In addition to improving temporal matching, the ability to simultaneously acquire both SPECT and CT modalities may provide modest improvements in efficiency with regard to image acquisitions. This is achieved by the elimination of typical movements required to move and reset the gantry and bed between modalities. Although the primary time limiting factor for any SPECT-CT study is generally the SPECT data collection, eliminating unnecessary inefficiencies in image acquisition can potentially reduce times significantly for animal studies involving a large number of subjects.

**Figure 1:**
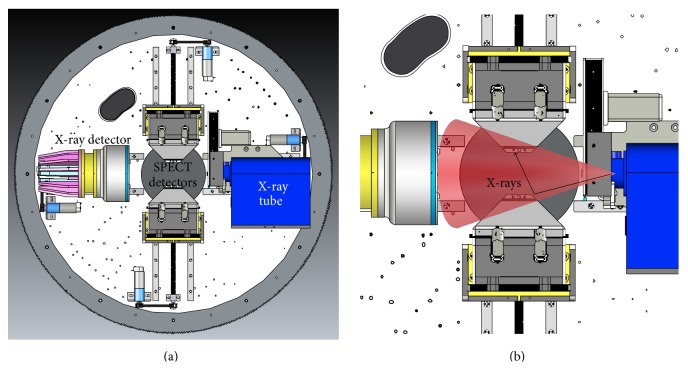
(a) The standard SPECT-CT hardware configuration for the Inveon platform and (b) illustration of the X-ray beam and photon scatter into the SPECT collimators.

Most* in vivo* preclinical micro-CT systems have an X-ray tube and detector mounted to a rotating stage as shown in Figure 1(a). The X-ray tube and detector are rotated about the object positioned in the center of the field of view (FOV) while projections are acquired at specified angles for a length of time determined by the exposure time. This method of acquisition is known as step and shoot and enables the high resolution imaging capabilities required of preclinical imaging systems at the expense of increased scan times [3]. SPECT imaging is performed in a similar fashion with detector heads mounted on a rotating stage and photons (or counts) from a radioactive source collected at specified angles around the object in the FOV. Preclinical SPECT detectors are often fitted with various collimator designs, such as multi-pinhole collimators, that enable higher resolution imaging while maintaining sensitivity performance [4]. More in-depth information regarding preclinical SPECT and CT systems can be found in* Small Animal Imaging* [5] and* Molecular Imaging: Principles and Practice* [6].

This work examines the feasibility of modifying a commercially available small animal SPECT/CT platform and assesses key performance parameters and limitations related to such modifications. Modification of existing scanner systems to offer such technology may provide a cost effective way of improving small animal SPECT/CT imaging workflows without the need to buy additional specialized hardware.

## 2. Materials and Methods

### 2.1. Imaging Platform

The SPECT-CT system modified for this work consisted of two SPECT detectors: an X-ray detector and an X-ray source mounted coaxially on a rotating gantry [7]. Each of these components is mounted on high-precision, computer-controlled motion stages for automatic FOV adjustment. The X-ray source operates in a voltage range of 30 to 80 kVp, while the SPECT detectors operate within an energy range of 30 to 300 keV. The SPECT data were acquired in listmode format that enabled the energy window to be selected after acquisition rather than having to know the optimum energy window prior to beginning the scan [8].

An Inveon SPECT-CT system (Siemens Medical Solutions USA, Inc.) was modified to support simultaneous acquisitions with the intent of performing this task with minimal hardware or software modification. This particular unit has a number of motion sensors that detect the movement of the X-ray and SPECT detector components in order to prevent possible collisions as the components move towards the center of the gantry to reach their default imaging positions. Manufacturer precautions do not allow the CT and SPECT subsystems to be used simultaneously at their default imaging positions as the components would potentially collide as they meet towards the center of the gantry. Disabling these precautionary mechanisms enables the SPECT and CT components to be simultaneously moved in towards the center of rotation while also allowing custom positioning of each of the components so that the fields of view of each subsystem can be optimized for simultaneous imaging without colliding.

Ideally, the SPECT and CT fields of view (FOVs) would be identical in simultaneous acquisition modes; however, this system has two X-ray detector options of an 8.4 cm × 5.5 cm or 10 cm × 10 cm detector. The active CT FOV depends on the detector size and the magnification setting (source to detector relative position). The SPECT FOV varies as well depending on the collimator chosen and the magnification settings. In order to best match the simultaneous acquisition FOVs, the X-ray CT components were placed in a medium magnification mode that provided a magnification factor of approximately 1.9. In this configuration, the achievable spatial resolution of the CT is 40 to 200 microns, depending on the X-ray detector binning factor. With the larger area detector (10 cm × 10 cm) installed in this gantry, the maximum axial FOV that could be matched to the SPECT system was 6.7 cm. However, the axial FOV can be extended by scanning with a helical orbit during acquisition.

### 2.2. Simultaneous SPECT-CT Initial Testing and Optimization

Before starting fully 3D SPECT/CT imaging, we tested the simultaneous acquisition configuration to determine how the two modalities would interact using two-dimensional imaging techniques. Radiographs and scintigraph images were taken using CT and SPECT subsystems, respectively, with a 1 mCi ^57^Co point source in the SPECT FOV and the CT kVp varied from 60 to 80 kVp. This was performed for each kVp setting to examine potential effects of X-ray scatter and pulse pileup through the collimators of the SPECT subsystem during simultaneous acquisition.

**Figure 2:**
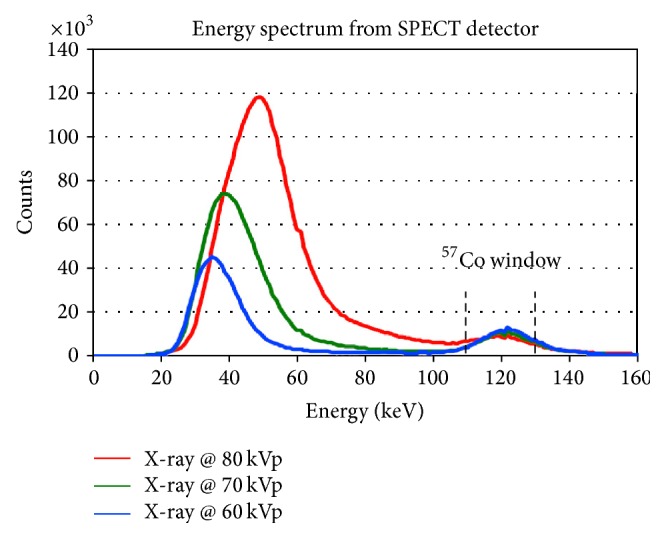
Spectrum collected from the SPECT detectors with varying kVp settings and a ^57^Co source present.

Scintigraphs were acquired for each setting with a data acquisition time of 100 s per projection. The emission data were histogrammed using a wide open window with a range from 0 to 300 keV and also a 20% energy window around the ^57^Co photopeak (122 keV) with a lower bound of 110 keV and an upper bound of 134 keV.  Figure 2 shows the spectrum acquired from the SPECT detector with varying X-ray voltage with a ^57^Co source present.

These projection data were used to create plots of measured counts versus X-ray tube voltage. The data were analyzed to find the X-ray tube setting where the measured counts with the X-ray tube on and no ^57^Co source in the FOV was approximately equal to the measured counts with only the ^57^Co source in the FOV. Since the X-ray tube and SPECT detectors are orthogonally oriented with no X-ray photons directly incident on the SPECT detectors, this crossover point would be the maximum X-ray tube kVp setting that would provide the greatest possible X-ray flux for CT imaging with the least count contamination of the SPECT data. These results were used as the optimum configuration of the X-ray tube for all subsequent simultaneous SPECT/CT protocols.

### 2.3. Acquisition and Postprocessing Modifications

The SPECT system in this platform uses a helical acquisition orbit. The CT subsystem was only capable of circular orbits (no bed travel during gantry rotation) when using the software provided by the manufacturer. The CT subsystem was modified to also acquire data helically to match the primary SPECT acquisition mode and to enable acquisitions with ideal axial sampling, better matched fields of view and ultimately simultaneous acquisition of SPECT and CT data. In brief, the user was given the ability to specify a distance to move the bed pallet axially between each CT projection.

An additional issue that arose with acquiring simultaneous SPECT-CT data in this gantry was that the center of the CT FOV was mechanically positioned approximately 18 mm axially from the center of the SPECT FOV. This issue could not be resolved without significant hardware modifications to the SPECT and CT subsystem mounting hardware, which was outside our constraints of minimal modification. This limitation simply meant that images acquired during simultaneous SPECT-CT scans covered slightly different regions along the axial direction of the phantom with the loss in coregistered FOV being equal to the offset between the two imaging subsystems (18 mm).

This SPECT-CT platform has two computers that comprise the data collection architecture. An embedded computer that is internal to the gantry controls all commands to the hardware while an external workstation, or host computer, sends requests to the embedded computer via a Gigabit Ethernet connection. This architecture was exploited in this study by forcing simultaneous acquisition of data by performing independent acquisitions on the two separate computers simultaneously. During tomographic scans, SPECT data was acquired by the embedded computer while the X-ray CT data was collected using the host computer, which defined the gantry and bed motion for the imaging protocol.

The postprocessing software had to be modified to properly histogram the SPECT listmode data. The SPECT data were reconstructed with the manufacturer's software unaltered. CT only data acquired with circular gantry orbits were reconstructed using a dedicated workstation with COBRA high speed reconstruction software (Exxim Computing Corporation). Helical CT data (acquired during simultaneous SPECT-CT scans) were reconstructed using an implementation of a 3D weighted FBP algorithm for spiral CT as described by [9, 10].

### 2.4. Simultaneous SPECT-CT Tomographic Acquisitions

CT data acquisitions were performed using a voltage of 70 kVp (found from initial planar studies) and anode current of 500 *μ*A. CT only scans were acquired with 360 projections over a full 360-degree rotation. For each CT projection an exposure time of 0.25 s was used. For the Derenzo phantom studies, contrast was enhanced by dissolving iodized salt into the water prior to imaging with CT. This provided better visualization of the Derenzo phantom rods with the CT modality.

The radius of rotation for the SPECT detectors was set to 50 mm resulting in a 100 mm bore size and a transaxial FOV of roughly 6.6 cm. SPECT data were acquired with 120 and 180 projections over 360 degrees with per projection acquisition times of 7 to 30 seconds, depending on the study and radioactivity in the phantom. This resulted in total scan times of 15 minutes for the uniformity phantoms and 30–40 minutes for Derenzo phantoms imaged using SPECT/CT. An additional 72-minute SPECT only scan was performed on the Derenzo phantom to acquire a high count dataset for comparison. It is important to note that although scan times varied, the object was only exposed to incident radiation from the CT X-ray tube for no more than approximately 90 seconds during the course of the entire scan.

The ^99m^Tc phantoms used for this study were a uniform water phantom with a 30 mm diameter and a Derenzo phantom (Micro Hot Spot Phantom, Data Spectrum Corp.) with rod sizes of 4.8, 4.0, 3.2, 2.4, 1.6, and 1.2 mm. The uniform syringe contained an aqueous solution of ^99m^Tc with a total activity of 1.8 mCi at scan time. The Derenzo phantom was injected with approximately 3 mCi of ^99m^Tc. For each series of experiments, the number of total counts in each scan was kept as similar as possible for the scans lasting 30–40 minutes by increasing the necessary per projection acquisition time to compensate for the natural decay of the ^99m^Tc isotope.

For each of the ^99m^Tc phantom studies, data were acquired in the following modes: SPECT only, CT only, and simultaneous SPECT-CT. The CT data were examined to verify that the SPECT isotope in the FOV during the CT did not cause any image artifacts or errors in quantitative measurements from the CT modality. The collimator used for SPECT acquisition of the ^99m^Tc phantom data was a 3-pinhole rat whole-body tungsten collimator with 1.2 mm diameter pinholes. This collimator was chosen because it provides spatial resolution sufficient for animal imaging with the largest FOV possible on this SPECT platform, providing the greatest potential SPECT-CT coscan range. The CT data were reconstructed using a 0.13 mm voxel size. All SPECT data were reconstructed with an implementation of 3D-OSEM using a model of the system point-spread function with 8 iterations, 12 subsets, and a voxel size of 0.5 mm [11]. Regions of interest were drawn on the SPECT only, CT only, and SPECT-CT data and statistical difference between values assessed with *p* < 0.05 assumed to be significant. The same region of interest was used for each measurement to ensure that the statistics for the ROI and the dimensions were kept constant between each individual measurement.  Figure 7 shows the uniform syringe phantom with example regions of interest used for the CT, SPECT, and SPECT-CT data.

### 2.5. Data Analysis

**Figure 3:**
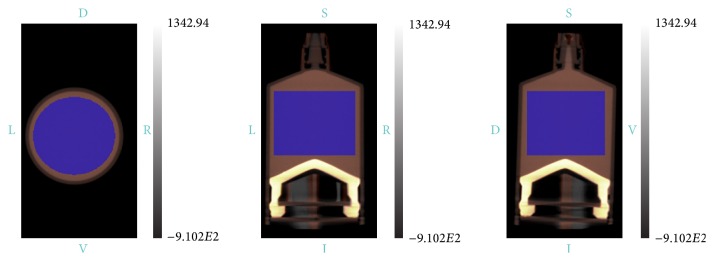
The region of interest drawn for uniformity measurements.

Uniformity analysis on the cylinder phantoms was performed by drawing a single rectangular region of interest (ROI) with a volume of 10,675 mm^3^ using the Inveon Research Workplace (IRW) software version 3.0 (Siemens Medical Solutions USA, Inc.). This ROI was positioned centrally in the transaxial and axial directions within the phantom. Mean to standard deviation calculations were made in spreadsheet software as well as calculations of the signal to noise ratio using the SNR equation below. Regions of interest were drawn on the SPECT only, CT only, and SPECT-CT data. The same region of interest was used for each measurement to ensure that the statistics for the ROI and the dimensions were kept constant between each individual measurement.  Figure 3 shows the uniform syringe phantom with example regions of interest used for the CT, SPECT, and SPECT-CT data:

(1) $$\begin{array}{c} \text{SNR}=\frac{\mu_{\text{signal}}}{\sigma_{\text{signal}}}. \end{array}$$

Tables were generated for the regions drawn on the CT only, SPECT only, and the SPECT-CT data. Measured ROI values and comparisons between experiments are shown in the Results. Confidence intervals (CI) were calculated to assess difference between the scans. For this small population, overlapping CI and *p* > 0.05 were assumed to be a nonsignificant difference between samples.

**Figure 4:**
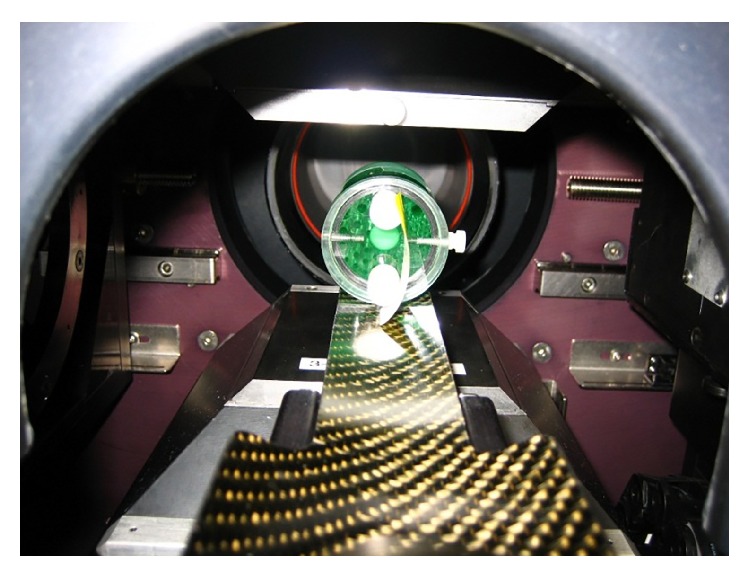
Image of the ^99m^Tc and saline filled Derenzo phantom on the carbon fiber imaging pallet provided by the manufacturer.

**Figure 5:**
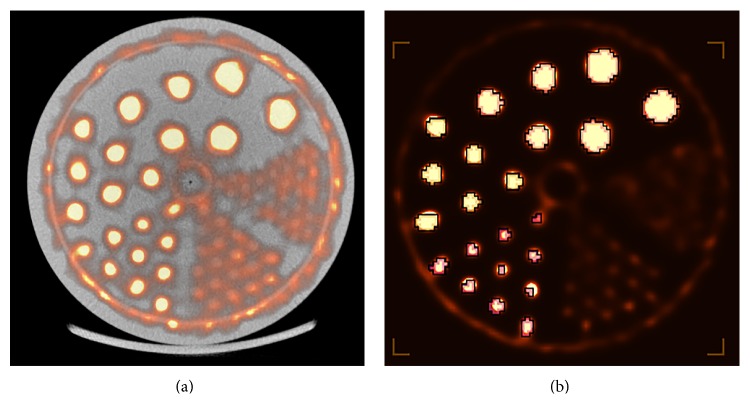
(a) Simultaneous SPECT-CT image of the Derenzo phantom and (b) regions of interest drawn on the SPECT data using a threshold of 40% maximum voxel value.

Visual analysis of the Derenzo phantom scans was performed using IRW to determine whether there is significant degradation in the SPECT image quality between X-ray on and off images. Using IRW to draw line profiles along the *y*-axis of the central axial view and ROIs over the phantom rods, we examined peak-to-valley ratios, standard deviation to mean values, SNR, and 95% CI within the rods to determine if and how much degradation in image quality might occur in simultaneous SPECT-CT measurements.  Figure 4 shows a photograph of the Derenzo phantom loaded into the SPECT-CT system, and Figure 5(a) shows a reconstructed simultaneous multimodal image of the Derenzo phantom using SPECT with contrast-enhanced CT.  Figure 5(b) illustrates the ROIs drawn within the hot rods used for the quantitative analysis of each of the Derenzo phantom scans.

## 3. Results

### 3.1. Initial Optimization

**Figure 6:**
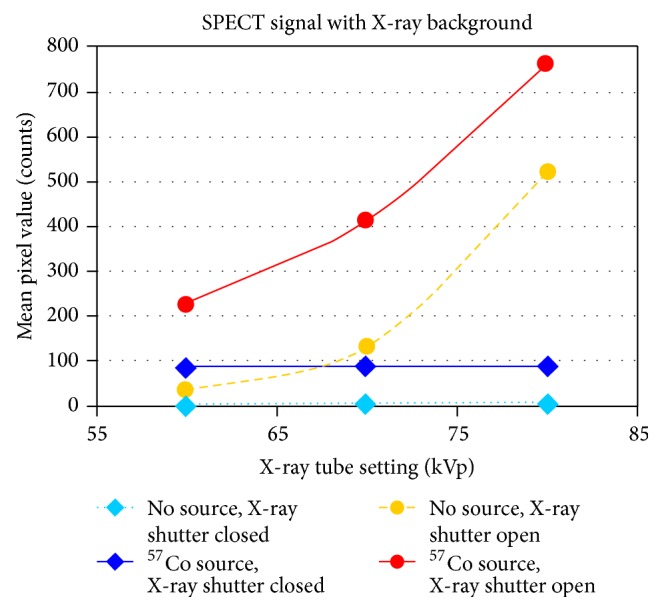
Plot of mean pixel values in SPECT projection data versus X-ray tube voltage used for verification of X-ray kVp setting. SPECT energy window settings were 110–134 keV.

**Figure 7:**
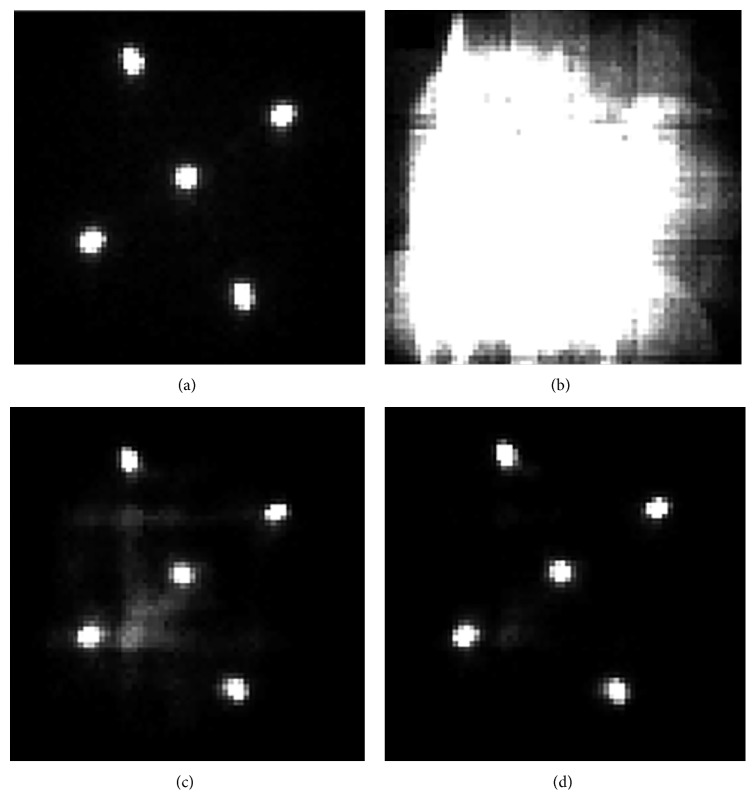
SPECT projections of a ^57^Co point source using a 5-pinhole tungsten collimator. (a) 30–300 keV energy window and no X-rays present, (b) 30–300 keV energy window and X-ray source on 80 kVp, (c) 110–134 keV energy window and X-ray source on 80 kVp, and (d) 110–134 keV energy window and X-ray source on 70 kVp.

Figure 6 shows the plot of the counts measured in the SPECT projection data as the X-ray tube voltage was swept from 80 to 60 kVp in the absence and presence of a ^57^Co point source. At 70 kVp, the mean counts from the orthogonally oriented X-ray source are nearly equivalent to those from a point source located in the center of the FOV. The 70 kVp X-ray tube setting was used for all subsequent simultaneous imaging tests with further proof of this optimization shown in Figure 7. Note that the curves do not sum linearly because when the ^57^Co source is present in the FOV, it scatters additional X-rays into the SPECT detectors.

In Figure 7(a), an image of a ^57^Co point source in the central FOV is shown with no X-rays present from the CT system and a wide open energy window of 30–300 keV. In this figure, the single point source appears as five individual sources because of the 5-pinhole collimator used to acquire the projection data.

Data acquired using a wide open energy window with the ^57^Co point source in the FOV and with the X-ray source on and at full power (80 kVp, 500 *μ*A) showed obvious scatter and pulse pileup effects. In this image it was impossible to discern the individual ^57^Co point source in the projection data. These effects on the SPECT projection image were severe, as seen in Figure 7(b). Adjusting the energy window to the standard 20% imaging window (110–134 keV) used for ^57^Co imaging showed a dramatic improvement in planar image quality even with full power X-rays present; however, some significant artifacts remained in the project data (Figure 7(c)). Data using the standard 20% ^57^Co imaging window and the optimized 70 kVp X-ray tube settings resulted in relatively clean projection images (Figure 7(d)) acquired in a simultaneous manner with these fully optimized results used for subsequent testing.

### 3.2. Simultaneous SPECT-CT Acquisition

The results in Table 1 show that the CT image uniformity was not adversely affected by the presence of radioactive ^99m^Tc with no statistical difference between mean HU values (*p* > 0.05). Mean values in the phantom varied by only 7% with overlapping 95% confidence intervals. The uniformity phantom SNR of the syringe filled with water was 3.88, while the uniformity of the syringe filled with ^99m^Tc solution was 3.84, a minimal 1.03% decrease in uniformity.

The measurements from the SPECT only scan (no X-rays) and the simultaneous SPECT-CT scan (with X-rays) are shown in Table 2. These measurements also yielded no significant difference in mean count values with *p* > 0.05 and a difference in counts of 28%. SNR also showed a minimal 12% difference which was nearly 47% less than the standard deviation to mean ratio for mean counts.

**Figure 8:**
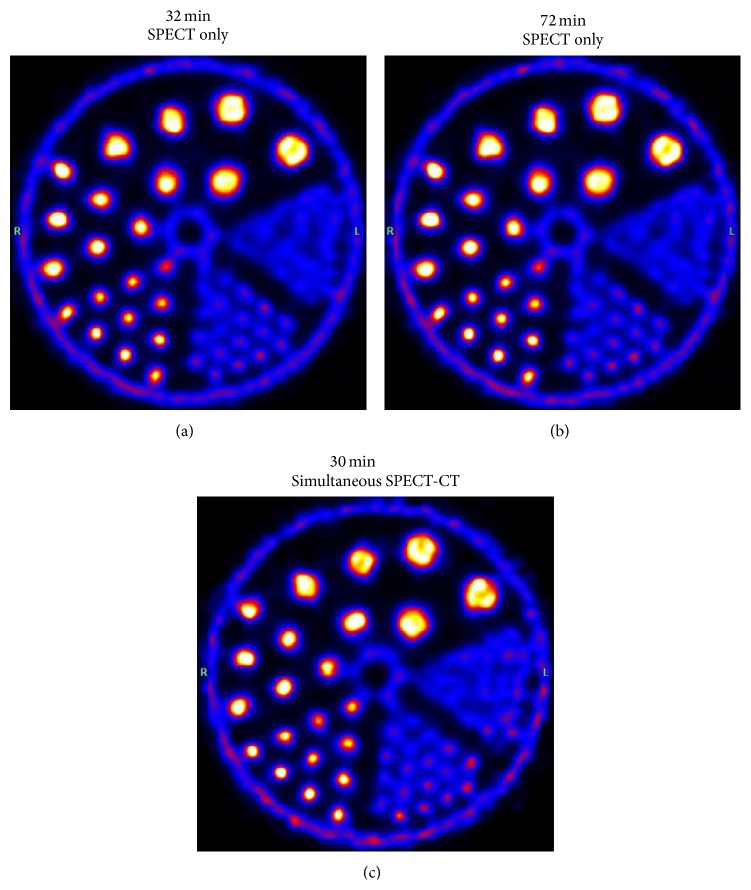
A comparison of SPECT only and simultaneous SPECT-CT images. (a) shows a 37-minute SPECT only, (b) shows a 72-minute SPECT only, and (c) shows SPECT data from a 30-minute simultaneous SPECT-CT workflow. Rod diameters are 4.8, 4.0, 3.2, 2.4, 1.6, and 1.2 mm. All window/level settings were set to display a range of 0 to 40% of the maximum value.

Visual inspection of the SPECT/CT Derenzo phantom data, shown in Figure 8, showed little degradation in image quality and spatial resolution.  Figure 8(a) shows a 37-minute SPECT only acquisition.  Figure 8(b) is a SPECT only study with an acquisition time of 72 minutes, representing a best-case scenario. Finally, Figure 8(c) shows the Derenzo phantom imaged by a 30-minute simultaneous SPECT-CT workflow. A slight negative impact on image quality and resolution appeared to occur in the simultaneously acquired SPECT image.

### 3.3. Line Profile Assessment

**Figure 9:**
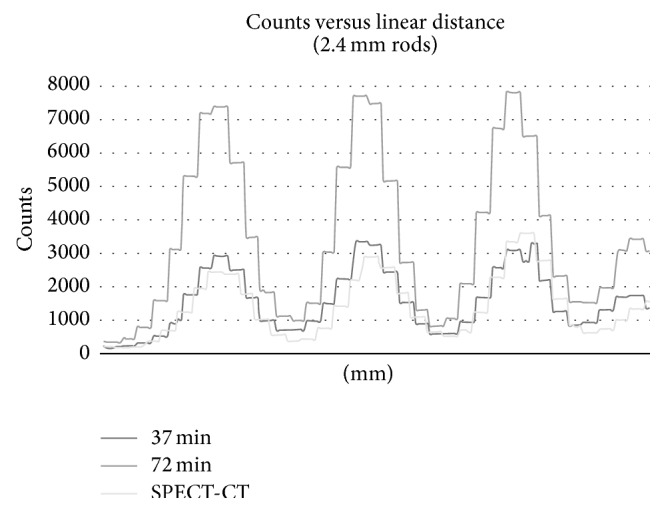
The figure shows a line profile drawn across the 2.4 mm hot rods.

The peak-to-valley ratios for each profile drawn on the various rod sizes show consistent ratios for the 37-minute SPECT, 72-minute SPECT, and the SPECT-CT scans, with percentage difference from the average values never reaching more than 8% for the SPECT-CT studies. Additionally, the ratios of peak-to-valley measurements between decreasing rod sizes also show consistent reductions indicating that the simultaneous SPECT-CT acquisitions are not significantly degraded compared to the SPECT only workflows.  Figure 9 shows a plot of the line profile drawn across the 2.4 mm rods for the SPECT only and SPECT-CT acquisitions. Tables 3 and 4 present the average peak-to-valley ratio measurements for each rod size as well as calculations of the ratio of peak-to-valley between rod sizes.

### 3.4. Region of Interest Analysis

In regions of interest drawn on the hot rods of the Derenzo phantom, signal to noise ratio in the rods improved with simultaneous imaging contrary to the initial visual assessment. The mean values for the 37-minute and 72-minute SPECT acquisitions are consistent as the mean number of counts in the segmented regions increases proportionally with scan time (approximately 2x). The simultaneous SPECT-CT acquisition also shows agreement with this expected result as the ratio of mean counts in identical regions is less by a factor of 1.2.  Table 5 shows the full ROI statistics as well as the signal to noise ratio and standard deviation to mean calculations.

## 4. Discussion

The data indicate that the Inveon SPECT-CT can be used for simultaneous SPECT-CT imaging. Good results were obtained both for a uniform phantom with known activity concentrations and the Derenzo phantom used for image quality assessments. For each series of measurements using the empirically derived parameters, the results indicated minimal degradation of image quality when simultaneous SPECT and CT acquisition was performed for the isotopes examined.

This is an important development since all imaging on preclinical systems is performed serially. Although work in the area of simultaneous detection and cross-talk correction methods have been performed [12, 13], none of this research looked at the possibility of converting a commercially available unit for the purposes of simultaneous imaging. The ability to convert a readily available imaging system means a greater likelihood that other researchers could use this work where previous research systems are only accessible by those labs that created them.

Our work can potentially be used to improve synchronization between modalities when performing complex imaging workflows such as those required for cardiac and respiratory gated acquisitions. With sequential acquisition, it becomes more likely that unwanted variations in physiology will occur between modalities. Simultaneous acquisition reduces this variation as each projection acquired for SPECT and CT were acquired during the same general time in the animal's physiology. Improvements in gating capabilities using low-dose detector technology may also improve gating applications with significantly shorter exposure times [14].

Testing indicated that the highest useful X-ray setting that could be used simultaneously with SPECT imaging on this platform was 70 kVp. Projection data acquired with X-ray energies above 70 kVp showed significant artifacts that are most likely due to pileup effects and some septal penetration of the collimator. Although not directly visualized, X-ray photons falling outside the energy window settings could result in dead time issues if the photon flux was sufficient. Because of the orthogonality of the CT and SPECT components, the estimated counts per second were not substantial enough to cause deadtime issues on this system.

Simultaneous acquisition also means potential improvements in coregistration between data. If the subject moves in one modality then it also has moved in the other. This may provide more accurate attenuation and scatter corrections and more quantitatively accurate data. Many studies have been performed to assess the effects of misregistration of correction data which result in incorrect voxel values in the regions of movement [15].

During this work, we also realized some small improvements in scan time. Only a small number of slip ring CT systems exist for small animal imaging which limits the gantry rotation speed of small animal SPECT/CT platforms. This results in fairly large percentages of the total CT acquisition time being the physical movement of components. Simultaneous acquisition can improve this as the gantry potentially needs only to rotate once to complete the acquisition of both modalities as shown by Austin et al. in the supplementary materials [16].

Additional time savings were realized by implementing helical CT. On this step and shoot platform, extending the axial range may result in multiple bed positions being acquired. This increases the scan time linearly with the number of bed positions. By using a helical CT to match the SPECT acquisition this removes the necessity for multiple bed imaging leading to improved total scan times.

Although simultaneous imaging has some unique benefits, it is not without compromise. On this particular imaging platform, the primary limitation is the maximum achievable SPECT resolution. Because of the hardware positioning within the gantry, the minimum distance between the SPECT detector and the subject is increased compared to the standard hardware configuration resulting in a reduced maximum resolution. The collimators used to image the Derenzo phantom in this study are specified by the manufacturer to achieve a maximum spatial resolution of 1.4 mm FWHM. Derenzo phantom images presented in this work showed that even in this reduced resolution configuration rod diameters between 1.6 and 2.4 mm were clearly visible. This slight decrease in resolution shows that rat imaging applications would not be significantly affected nor would mouse imaging protocols, such as screening, where this decrease in resolution would only minimally impact the data. Protocols requiring high resolution imaging would certainly be degraded using a simultaneous technique on this particular commercial platform.

There appeared to be a slight reduction in image quality and image resolution for simultaneously acquired data. This was most likely caused by the introduction of scattered X-ray photons and pileup effects. Resolution reductions were seen as a reduction in the separation between the smallest visible rods in the Derenzo phantom. This loss may have been caused by reduction in overall contrast during the simultaneous SPECT-CT scan.

The wide energy gap between ^99m^Tc photon emission (140 keV) and the average X-ray photon energy (25–35 keV) make separation of the counts easier. For isotopes with emissions that overlap the range of X-ray energies, such as ^125^I (25–36 keV), separation of the individual contributions could be more challenging. Although this is a limitation for this technique, low energy SPECT isotopes are not used as frequently as higher energy isotopes such as ^99m^Tc and ^123^I.

Future work in simultaneous acquisition methods could include development of novel ways to perform simultaneous imaging with ^125^I and to assess any specific sensitivities that might exist with this isotope. The primary issue would be to develop a method by which the gammas emitted from ^125^I could be separated from any CT X-rays. One approach would be to use a Monte Carlo model that would enable a statistical method of removing the X-rays from the ^125^I SPECT projection data, such as the GATE model created for this platform by Lee et al. [17, 18].

For complete validation of this method, animal studies would be necessary to fully validate this method. Animal acquisition was not possible for this study because of facility limitations where this research occurred. This work does provide the necessary tools for this to be repeated at a facility where access to animals for* in vivo* animal studies could be performed.

## Figures and Tables

**Table 1:** Region statistics for CT images with and without isotope present.

CT syringe	Mean (HU)	StdDev (HU)	95% CI	SNR	% Diff SNR (%)
H2O	18.6	4.8	9–28	3.88	
^99m^Tc	17.3	4.5	8–26	3.84	1.03

**Table 2:** Region statistics for SPECT only and simultaneous SPECT-CT uniformity measurements.

^99m^Tc syringe	Mean (cts/cc)	StdDev (cts/cc)	95% CI	SNR	% Diff SNR (%)
No X-rays	882.4	243.3	406–1359	3.63	
With X-rays	1130.4	277.9	586–1675	4.07	12.12

**Table 3:** Peak-to-valley ratios for each profile drawn through the rod phantom.

4.8 mm and 4.0 mm rods					
37 min SPECT	% Diff. of mean	72 min SPECT	% Diff. of mean	SPECT-CT	% Diff. of mean
25.93	0.6	25.75	1.28	26.57	1.88

3.2 mm rods					
37 min SPECT	% Diff. of mean	72 min SPECT	% Diff. of mean	SPECT-CT	% Diff. of mean

15.05	7.65	12.58	9.96	14.3	2.31

2.4 mm rods					
37 min SPECT	% Diff. of mean	72 min SPECT	% Diff. of mean	SPECT-CT	% Diff. of mean

6.23	19.51	8.65	11.85	8.33	7.65

**Table 4:** Ratios of peak-to-valley calculations.

Ratios between decreasing rod sizes				
	37 min	72 min	SPECT-CT	Average of all scans
4.8, 4.0 to 3.2 mm	1.72	2.04	1.86	1.87
3.2 to 2.4 mm	2.43	1.45	1.72	1.87

**Table 5:** Derenzo phantom hot rod statistics and SNR.

	Mean (cts/cc)	StdDev (cts/cc)	Min (cts/cc)	Max (cts/cc)	SNR (SNR)	SD/Mean (%)
37 min SPECT						
All rods	3466.9	923.1	2211.4	6619	13.6	26.6
4.8, 4.0 mm	3713.8	940.4	2211.4	6563.4	13.3	25.3
3.2 mm	3480.8	907.9	2211.4	6619	13.7	26.1
2.4 mm	2824.3	468.8	2211.4	4868.5	15.1	16.6

	Mean (cts/cc)	StdDev (cts/cc)	Min (cts/cc)	Max (cts/cc)	SNR (SNR)	SD/Mean (%)

72 min SPECT						
All rods	7011.3	2015.4	2114.6	13220.5	14.8	28.7
4.8, 4.0 mm	7580.2	2022.6	2801.7	13220.5	14.2	26.7
3.2 mm	7062.9	1913.6	2490.6	12987.9	14.8	27.1
2.4 mm	5546.3	1121.5	2573.5	8825.3	14.9	20.2

	Mean (cts/cc)	StdDev (cts/cc)	Min (cts/cc)	Max (cts/cc)	SNR (SNR)	SD/Mean (%)

30 min SPECT-CT						
All rods	2861.7	872.3	787.3	5501.5	14.7	30.5
4.8, 4.0 mm	3128	865.6	946.1	5501.5	14.4	27.7
3.2 mm	2851.6	824.3	869.5	5447.6	14.9	28.9
2.4 mm	2190.5	494.8	787.3	3970.7	16.2	22.6
